# Associations between Perceived Social Eating Norms and Initiation and Maintenance of Changes in Dietary Habits during the First COVID-19 Lockdown in France

**DOI:** 10.3390/foods10112745

**Published:** 2021-11-09

**Authors:** Armelle Garcia, Suzanne Higgs, Anne Lluch, Nicolas Darcel, Olga Davidenko

**Affiliations:** 1 INRAE, UMR PNCA, AgroParisTech, Université Paris-Saclay, 75005 Paris, France; nicolas.darcel@agroparistech.fr (N.D.); olga.davidenko@agroparistech.fr (O.D.); 2 School of Psychology, University of Birmingham, Birmingham B15 2TT, UK; S.HIGGS.1@bham.ac.uk; 3 Danone Nutricia Research, Centre Daniel Carasso, 91767 Palaiseau, France; anne.lluch@danone.com

**Keywords:** social norms, food consumption behaviour, COVID-19

## Abstract

Changes in dietary habits of the French population have been reported during the national lockdown that was enforced due to the COVID-19 pandemic. This study investigated whether perceived social eating norms were associated with the initiation and maintenance of dietary changes that took place as a result of lockdown. An online study collected information on (1) changes in consumption implemented during the lockdown and the maintenance of these changes, and (2) perceptions about changes in consumption implemented during lockdown by household members, relatives out of home, and the general population. The changes in consumption were classified as foods to increase or to decrease according to French national recommendations. The perception of changes to dietary habits by household members and relatives out of home was related to the changes made by individuals for each of the food categories (all *p* < 0.05) but not to the perception of changes made the general population. Increased consumption of foods to increase was more likely to be maintained when there was a positive perception of the changes made by household members (*p* = 0.03). These results highlight the influence of the perception of social eating norms, especially by household members and relatives, on the implementation of dietary changes during lockdown and suggest that social eating norms can have a lasting influence.

## 1. Introduction

To reduce the spread of the novel coronavirus SARS-CoV-2, France enforced a national lockdown between 17 March and 10 May 2020. It has been reported that the dietary habits of the French population changed during this period. Two opposite tendencies were highlighted by an online study conducted on a cohort of 37,252 French adults [1]; for some individuals, the nutritional quality of the diet decreased, while it increased for others. The change in nutritional quality of the diet seemed to depend on different individual (weight status and diet quality before lockdown, and anxiety) and contextual factors (sociodemographic and economic position, professional situation, and having child at home) [1,2]. Similar tendencies have also been reported in other countries such as the USA, Canada, Italia, Spain, Norway, UK, and Poland [3,4,5,6,7,8,9,10,11,12,13]. Decreases in nutritional quality were found to be associated with an increase in stress, anxiety, and a greater influence of mood on food choices during lockdown [6,9,14,15]. Regarding increases in nutritional quality, it seems that the pandemic may have enhanced awareness about the importance of dietary habits for health and environment and may have acted as a trigger for change for individuals who were already thinking of modifying their diet for health, environmental, or even ethical reasons [16]. Whether these changes to dietary habits are maintained or not could have long-term health effects.

Our dietary habits are determined by both individual and environmental factors. Individual factors include factors such as nutritional state, taste, stress, and motivations [17]. Environmental factors include the physical and the social environment in which we make our decisions [17,18,19]. It has been argued that living in a stable context leads to strong context–response associations, which results in the establishment of strong dietary habits [20]. By modifying the usual environment of individuals, the lockdown provided an occasion to establish new habits [21,22]. The lockdown also disrupted social interactions, and it is well known that social context has a profound influence on food intake and food choices [23,24,25,26,27].

The behaviour of other diners at mealtimes can serve as a social eating norm that provides a guide as to appropriate food choices [24]. Social eating norms can also be communicated through social media and TV, and such perceived social eating norms can have as much impact as the behaviour of others. For instance, at the beginning of the 2000s, many studies found that the majority of students had a biased perception of the norm of alcohol consumption of other students from their university, and that this biased perception had consequences for their own consumption. Specifically, a positive correlation was observed between perceptions of the amount of alcohol consumed by other students and the actual amount of alcohol consumed by participants [28,29,30]. Furthermore, additional studies demonstrated that, when the perception of alcohol consumption of other students was corrected, participants’ alcohol consumption habits changed to align with the new perception of the norm [31,32,33,34]. Similar effects have been reported for eating behaviours. For instance, correlations have been reported between the perception of social eating norms and the diet of participants [35,36]. Another study found that perceived social eating norms regarding the dietary habits of users of a social media site that was frequented by participants predicted their own consumption of fruit and vegetables [37]. Moreover, some studies have found that exposure to normative messages suggesting that fruit and vegetable consumption is the majority behaviour results in increased fruit and vegetable intake [38,39,40,41]. However, there has been little investigation of whether changes established in line with a new perception of social eating norms are maintained over time. Here, the specific context of the lockdown allowed us to examine the longer-term influence of perceived social eating norms on dietary habits. Our aim was to investigate whether perceived social eating norms are associated with both the initiation and the maintenance of dietary changes related to lockdown. Our first hypothesis was that the dietary changes initiated by participants would be significantly related to perceived social eating norms during lockdown (Hypothesis 1). We also hypothesised the perceived social eating norms during lockdown would be related to the probability of maintaining the changes after lockdown (Hypothesis 2).

## 2. Materials and Methods

### 2.1. Data Collection

**Figure 1:**
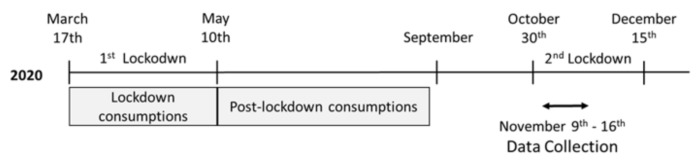
Timeline of lockdowns in France and data collection.

We conducted an online survey to assess self-reported food consumption and the perception of the consumption of others in French adults during the lockdown (17 March–10 May), and in the first months after lockdown (May–September). Participants were recruited through an online panel agency (EasyPanel) in November 2020 and were selected from a representative sample of the adult French population (probability sampling in term of gender, age, location, and profession) if they indicated having made changes to their dietary habits during the first lockdown. Data collection took place between 9 and 16 November 2020. At this time, a curfew was in place in France from mid-October, and the French population had just started a second lockdown (2 November–15 December). Therefore, the participants were asked to indicate their dietary habits during the first lockdown and after the lockdown, but only until September 2020 before the second lockdown came into force (see Figure 1 for timeline). The pandemic situation also explains why we decided to carry out this study using online questionnaires instead of physical interviews.

### 2.2. Survey

#### 2.2.1. Part 1

The first part of the survey, which was sent to a representative sample of the adult French population, included sociodemographic questions (age, sex, height, and weight, household composition, socio-professional category, and education level). Participants were also asked to state whether they had implemented any changes to their dietary habits (consumption, place of purchase, and cooking practices) during the first lockdown. As we were interested in the maintenance of changes and the factors influencing this maintenance, participants were only invited to complete the second part of the survey if they indicated a change in their dietary habits during the lockdown.

#### 2.2.2. Part 2

The second part of the survey included questions about the conditions in which participants spent the lockdown, the changes they made to their dietary habits and food choice motives, self-reported changes in food consumption as a result of lockdown, maintenance of changes in food consumption after lockdown, and perceptions about others’ consumptions during lockdown. These questions are detailed below.

- Lockdown Situation

The questions covering the lockdown conditions of the participant included the place of lockdown, household composition during the lockdown, financial situation, and time spent at home compared to before lockdown.

- Changes in Dietary Habits and Food Choice Motives

Participants were asked about changes to dietary habits: Was the lockdown an opportunity to rethink dietary habits (yes/no)? If yes, through what information sources (the Internet, household members, relatives out of home, social media, books, traditional media and television, movies and documentaries, podcasts)? If yes, did this rethink begin before lockdown (yes/no)? Were these changes voluntary (yes/no)? What is your opinion on these changes (positive or negative)? During lockdown, was the person responsible for cooking different from before (yes/no)?

The importance of 12 different food choice motives before, during, and after the lockdown was assessed (price, health, pleasure, mood, weight control, environment, geographical origin, animal welfare, availability, time of preparation, storage time, and risk of exposure to COVID-19 when shopping as people as a reason to modify purchase habits to avoid COVID-19 exposure). Participants were asked to rate the importance of these motives from 1 (not important) to 7 (very important). Motive scores during and after the lockdown were compared to motive scores before lockdown (∆ motive) to analyse whether they stayed the same (motive = 0), increased (∆ motive > 0), or decreased (∆ motive < 0).

- Self-Reported Changes in Dietary Habits as a Result of Lockdown

Participants were asked about how their consumption of different food items changed during and after lockdown compared to before lockdown (see Annex S2 for whole list of food items in the questionnaire). For the analysis, nine food items were used and were categorised into “foods to increase” and “foods to decrease” (Table 1), according to the French National Health Agency recommendations (Ministère des Solidarités et de la Santé, 2019). The French national recommendations contain a third category “foods to go towards”, which includes foods to favour such as whole grains, local foods, and organic foods, as well as food to consume in a limited quantity such as fish, seafood, and dairy products. This category was not included in our analysis because information was not collected on all the types of foods/practices in the category.

Changes in the consumption of each food item were coded as follows: never consumed, more, less, same. Numeric values were attributed to each food item (never = 0, more = +1, less = −1, same = 0) and cumulated for every item from the same category to obtain a total score for each category. If the total score obtained for a category was less than 0, then it was coded as “decrease”, if it was more than 0, then it was coded as “increase”, and if it was equal to 0, then it was coded “same”.

- Maintenance of Changes in Dietary Habits after Lockdown

For analysis of the maintenance of changes, only individuals who increased or decreased their consumption during lockdown compared to before lockdown were included. We compared self-reported consumption after lockdown compared to before lockdown to identify those who reported an increase or decrease in consumption and those who reported no changes in consumption compared to before lockdown. This allowed us to identify those participants who maintained any increase or decrease in consumption they reported during lockdown, as well as those who did not maintain these changes (see Table 2).

- Perceived Social Eating Norms

The survey included several questions that assessed the perceived social eating norms.

### 2.3. Social Eating Norms during Lockdown

The perception of three types of descriptive social eating norms during the lockdown was recorded. A descriptive social norm refers to how other people behave, in this case, whether they changed their dietary habits. Participants were asked to state if they were in lockdown with individuals who they thought made changes (*household social eating norm)*, and whether they believed that their relatives out of home (*non-household relatives eating social norm*) and the general population (*general population social eating norm*) made dietary changes during lockdown. Moreover, participants who perceived that others had made changes were asked to state whether they thought these changes were positive or negative. According to these answers, we then coded the social eating norms as categorical variables with three levels (yes positive/yes negative/no). Lastly, the participants were asked if they were aware of the influence of social eating norms on their own changes. For each social eating norm, individuals who stated that they believed that others had made changes were asked whether they thought that it influenced them in making changes or not. The entire survey is available in French in Annex S2. All participants gave their informed consent (see Annex S1). Ethical approval for the study was obtained from the ethics committee of Paris-Saclay University (registration number CER-Paris-Saclay-2020-078).

#### Statistical Analysis

The hypotheses and analytical plan were specified and preregistered before the data were collected (15 October 2020: https://osf.io/8gbk5/). *t*-Tests were used to compare participants who indicated that a change in their dietary habits during the lockdown (and, thus, who completed part 2 of the survey) with participants who indicated no change in their dietary habits (and, thus, only completed part 1 of the survey).

Multinomial logistic regressions were used to test the primary hypothesis. On the basis of the preliminary analyses which showed a strong correlation between the different types of social eating norms, we separately tested the effect of each type of social eating norm (household, relatives, and general population) on the reported consumption of food items in each food category (foods to increase and to decrease). Hence, we used six models for testing hypotheses 1 and 2. Each model was adjusted for sex, age, and BMI (the BMI was calculated from self-reported height and weight). The models were also adjusted for contextual covariates and changes in food choice motives [2] that could have explained the changes in consumption and the maintenance of these changes. Models examining the effect of social eating norms during lockdown on the implementation of changes (Hypothesis 1) were, thus, adjusted to take into account whether participants were in lockdown with somebody they do not usually live with (yes/no), changes in time spent at home (more/less/same), financial situation (easier/harder/same), change in the person responsible for meal preparation (yes/no), and change in the 12 food choice motives (more important/less important/same) during lockdown compared to before lockdown. Finally, the models examining the effect of social eating norms during lockdown on the maintenance of changes (Hypothesis 2) were further adjusted for whether or not the changes were voluntary (yes/no) and for changes in eating motives after lockdown compared to before lockdown (more important/less important/as important as before). R Studio version 1.1.463 [42] was used for data analysis.

## 3. Results

### 3.1. Sample Characteristics

A total of 1694 individuals from a representative sample of the French adult population completed the first part of the survey (909 women—54% and 785 men; mean age 47.6 years old (±14.8); mean BMI 25.1 kg/m^2^ (±4.9)). From this initial sample, 1008 individuals answered “yes” to the question *“Have you implemented any changes to your dietary habits during the first lockdown?”* and were invited to complete the second part of the survey (567 women—56% and 411 men; mean age = 45.6 years old (±14.9); mean BMI = 24.9 kg·m^−2^ (±4.7). These 1008 participants were included in the final analysis. They were on average significantly younger compared to excluded individuals (*n* = 686), and there was a higher proportion of women in the final sample. BMI did not differ between included and excluded individuals. The included population was composed of a significantly lower proportion of retired individuals and a significantly higher proportion of individuals working in executive positions compared to excluded individuals. Lastly, the included population was composed of individuals with a higher level of education compared to excluded individuals, with a significantly lower proportion of individuals with a professional diploma and a significant higher proportion of individuals with a master’s degree. See Table 3. for a complete comparison of included and excluded individuals.

The analyses reported below were carried out on the participants who confirmed changes to their diet during lockdown (*n* = 1008).

### 3.2. Lockdown Conditions

The majority of the participants stated that they were in lockdown in their primary residence (97%) and with persons they usually live with (90%). Half of the population stated that their financial situation during lockdown was equivalent to before lockdown (56%), whereas 28% stated that it was worse and 16% stated that it was better. One-third (33.3%) of the sample stated that they spent more time working at home during lockdown, falling to 18.8% after lockdown in September 2020. The vast majority of the population (87.5%) stated that there were no changes in the person responsible for cooking during lockdown.

### 3.3. Dietary Behaviours during Lockdown

#### 3.3.1. Changes in Dietary Habits and Motives

Half of the participants (51.6%) stated that the lockdown was an opportunity to rethink their dietary habits. Of these participants, 57.7% stated that these reflections had already started before lockdown. Different sources of information that influenced these reflections were, in order of importance, household members (42%), the Internet (40%), relatives out of home (38%), social media (21%), books (21%), traditional media and television (19%), movies and documentaries (13%), and podcasts (3%). The majority of individuals stated that the changes they had made were voluntary (81%), and 87% stated that they had a positive opinion of the changes made during lockdown. The importance of the following food choice motives increased during the lockdown: exposure to COVID-19, mood, product availability, and cooking time (see Annex S3 for full results on changes in motives).

#### 3.3.2. Self-Reported Changes in Dietary Habits as a Result of Lockdown

**Figure 2:**
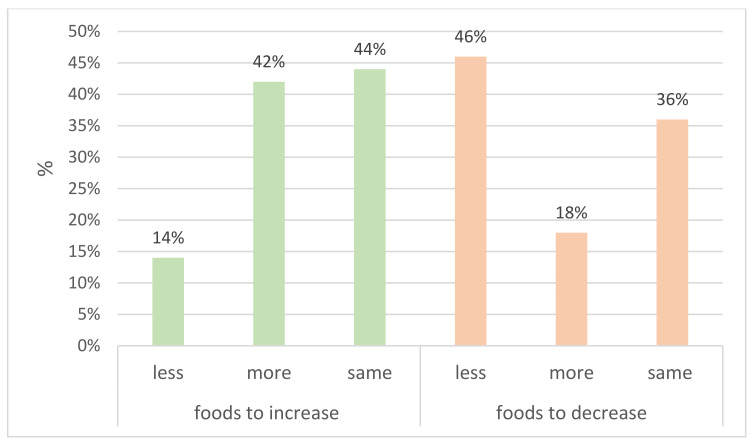
Changes in consumption per food category during lockdown compared to before.

Around 40% of our sample reported an equivalent consumption during lockdown compared to before. For foods to increase and foods to decrease, 42% reported making changes in line with recommendations by increasing their consumption of foods to increase, and 46% of the population reported decreasing their consumption of foods to decrease. Fifteen percent of the population made changes against recommendations, by decreasing their consumption of foods to increase and increasing their consumption of foods to decrease (see Figure 2).

#### 3.3.3. Maintenance of Changes in Dietary Habits as a Result of Lockdown

**Figure 3:**
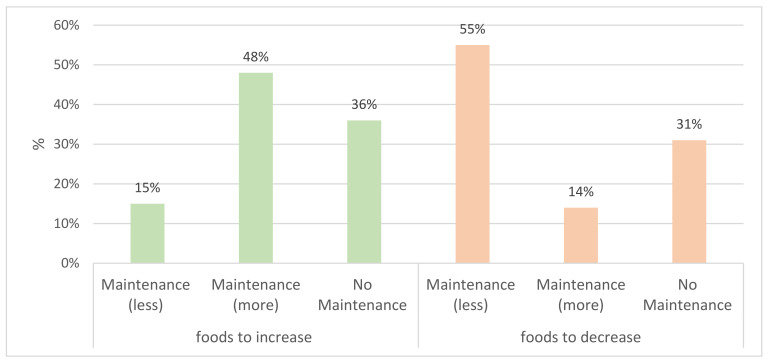
Maintenance of changes in consumption per food category after lockdown. *N* = 646 for foods to decrease; *N* = 568 for foods to increase.

Regardless of the food category, more than one-half of the participants who reported making changes during lockdown reported maintaining them after lockdown (63% and 69% for the changes of foods to increase and foods to decrease, respectively). More precisely, among the individuals who changed their consumption of foods to increase, 48% maintained an increase in consumption, and 15% maintained a decrease in consumption. Lastly, for the foods to decrease, 55% maintained a decrease in consumption, whereas 14% maintained an increase in consumption. Full results are presented in Figure 3.

### 3.4. Perceived Social Eating Norms during Lockdown

Thirty-nine percent of study participants stated that they were in lockdown with one or more individuals who had also changed their dietary habits (household social eating norm). The perceived changes in household members were regarded as positive for 37% of the participants and negative for 2%. Sixty-one percent of the population believed that their non-household relatives made dietary changes during lockdown (non-household relatives social eating norm), and 55% and 6% considered that these changes were positive or negative, respectively. Eighty-four percent of the population believed that the general population made dietary changes during the lockdown (general population social eating norm), with 70% stating that those were positive changes and 14% reporting that they thought that those changes were negative.

In addition, the majority of individuals who perceived that others had made changes during lockdown also stated that this influenced them in making their own changes (83.8% of individuals for the household social eating norm, 80.8% of individuals for the relatives social eating norm, and 71.3% of individuals for the general population social eating norm).

### 3.5. Hypothesis Testing

**Hypothesis** **1** **(H1).** *Association between lockdown perceived social eating norms and the initiation of changes.*

Multinomial logistic regressions revealed a significant relationship between perceived social eating norms about household members and non-household relatives changes and the changes initiated by the participant for each food category. For foods to increase, social eating norms about household members and non-household relatives that were perceived as positive were associated with an increased likelihood of participants increasing their consumption. Participants who perceived household social eating norm as positive also had a greater likelihood of decreasing their consumption of foods to decrease, while those who perceived household social eating norm as negative were more likely to have increased their consumption of foods from this category. Surprisingly, the perception of a household social eating norm as positive was also found to be significantly associated to an increase in consumption of foods to decrease. No significant associations between perceived general population social eating norms and changes in consumption were found for any of the food categories (see Table 4).

**Hypothesis** **2** **(H2).** *Influence of lockdown perceived social eating norms on the maintenance of changes.*

We found a significant relationship between the perception of a household social eating norm and the maintenance of changes, but only for foods to increase. More precisely, the perception of a positive household social eating norm regarding changes made during lockdown was associated with a greater likelihood of maintaining an increase of consumption of foods to increase. There was no significant association among perceived relatives social eating norms, the general population social eating norms, and the maintenance of changes, for any of the food categories (see Table 5).

### 3.6. Covariates

For detailed results of all covariates, from all the models, see the Supplementary Materials (Table S1).

## 4. Discussion

### 4.1. Effects of Social Eating Norms

The results from this study highlight a potential role for perceived social eating norms in the initiation and the maintenance of dietary changes that took place during the first lockdown in France. We found that the perception of changes made by household members and relatives out of home was significantly related to the changes made by the participants when controlling for other factors. The perception that the changes made by household members were positive was also related to the likelihood of participants having maintained dietary changes 5 months after the end of the lockdown.

We observed a significant relationship between the self-reported dietary changes made by participants and their perceptions about the dietary habits of others. In other words, participants who reported that they believed that others had made dietary changes during lockdown had a higher chance of making changes themselves. However, only the perceived changes made by household members and relatives, but not by the general population, were associated with the self-reported changes made by participants. These results are congruent with participants’ reported awareness of the influence of others’ behaviour on their own behaviour. A higher proportion of individuals said that the changes made by household members and relatives influenced them (respectively 83.8% and 80.8%) compared to changes made by the general population (71.3%). Lastly, 40% of our population reported that discussions with relatives at home and out of home prompted them to rethink their dietary habits. Together, these results suggest that, in line with previous research, the behaviour of familiar others has more impact than the perceptions about behaviour of the general population [43,44,45,46].

Our data also tentatively suggest that participants were more influenced by norms that they perceived as positive than norms that were perceived as negative. We observed that participants who perceived the changes made by others as positive were more likely to report making changes to their own diet that were in line with nutritional recommendations. At the same time, those who perceived changes made by others as negative were more likely to make changes against the recommendations, but only for the foods to decrease category. However, these results should be considered with caution because the majority of participants reported that their perception of dietary changes made by others were positive (respectively 93%, 90%, and 84% for household, relatives, and the general population social eating norms); therefore, there may have been less power to detect associations with negative social eating norm perceptions. In addition, social eating norm perceptions can be biased towards the individual’s own behaviour, which is known as social projection [47,48,49]. It is possible that participants had a biased perception of others’ changes, based on their own changes. Furthermore, the present study does not allow concluding on whether it is the perception of the norms or the actual norms that are underlying the associations we observed.

This study was an observational study, and we cannot say whether the observed association is due to a direct causal effect of social eating norms or due to a biased perception of others’ behaviour. It is also possible that the observed associations are due to direct effects of others making changes, especially for household members where the behaviour of one household member is susceptible to the direct influence other household members through practical changes such as common food purchases. However, the fact that this association was also found for non-household relatives indicates that this influence is not solely explained by such direct effects. Several individual (sex, age, and BMI) and contextual (being in lockdown with people we usually do not live with, difference in the person responsible for cooking, and changes in food choice motives) covariates were also associated with dietary changes and the maintenance of changes. Hence, the perception of social eating norms might not have been, on its own, responsible for changes established and their maintenance. Although the results of this study do not provide conclusive support for a direct causal effect of social eating norms on changes to dietary habits, previous studies have found that correcting the misperception of social eating norms can modify behaviour in accordance with the new perception of social eating norms [31,32,33,34,35,50,51]. Such results illustrate the influence of the social eating norms perception on behaviours and indicates that this direction of effect cannot be excluded.

Importantly, the present study is one of the first to suggest that social eating norms could have long-term effects, as we also found that perceived social eating norms were associated with the maintenance of changes 5 months after the lockdown. Interestingly, only the maintenance of changes in line with the recommendations were associated with social eating norm perceptions. Social eating norms perceived as positive may not only have a direct influence in the moment, but could also act as a reinforcement of positive behaviour, strong enough to have long-term effects. Studies have shown an activation of the brain reward networks when individuals conform to social eating norms [52,53]. It is, therefore, possible that positive emotions that took place when behaving in accordance with the norm increase the likelihood of changes being maintained over time [24]. However, this study took place in a very particular social context, and the results should be confirmed in a more normal context after the pandemic.

### 4.2. Main Dietary Changes Observed

We observed changes both in line and against nutritional recommendations, which is congruent with the results from other studies conducted during the pandemic [1,2,3,4,5,6,7,8,9,10,11,12,13,14]. In France, Marty and colleagues found a significant increase in the reported consumption of both foods to increase (fruits, vegetables, and pulses) and foods to decrease (processed meat, sweet-tasting beverages, sugary foods, alcoholic beverages, and salt), but they observed a global reduction in diet quality (sPNNS-GS2 score) [2]. In the present study, we did not examine changes in consumption for all food categories for each individual, which means that we could not assess general changes to diet quality.

### 4.3. Maintenance of Dietary Changes

This study is one of the first to examine the maintenance of changes to dietary habits during lockdown in France. We observed that, 5 months after the end of the first lockdown, around 65% of individuals who reported that they had made dietary changes also reported that they had maintained them. A study conducted in China also observed that healthy dietary behaviours implemented during the COVID-19 lockdown were maintained 5 months later [54]. Together, these results suggest that, according to self-report surveys, the changes established during the lockdown may persist in the longer term, but additional studies need to be done to confirm these observations.

In addition to the potential influence of social eating norms, we identified potential additional explanations for the maintenance of changes. We observed that a high proportion of participants stated that their changes were voluntary (81%) and that the lockdown was an opportunity for them to rethink their dietary habits (51.6%). One-third (30%) of the participants also stated that this reflection started before lockdown. These results suggest that the first lockdown might have acted as a transition accelerator for changes in dietary practices that individuals were already considering. Hence, it is possible that pre-contemplated changes have a higher chance of being maintained compared to more spontaneous changes.

We also observed that the household environment of most individuals during lockdown did not change greatly (97% of the population was in lockdown in their usual place of residence, and 90% of participants were in lockdown with people there are usually living with). As the changes were established in a familiar environment, which remained the same after lockdown, this might also have facilitated the maintenance of changes even after the lockdown. At the time the study was conducted (November 2020), the pandemic was not over, and social interaction levels were still not comparable with the period before March 2020. This implies that, even after lockdown, direct external influences from people we are eating with out of home were still reduced, which could have also facilitated the maintenance of changes established in a context with a reduced level of social interactions during lockdown.

Together, our results have implications for public health strategies aimed at enhancing diet quality. Several reports have concluded that the health communication strategies that have been implemented in Europe have not been sufficient to promote healthier eating patterns [55,56]. Although people may be aware of healthy eating recommendations, they do not always adhere them. According to the theory of planned behaviour [57], intentions govern the implementation of a behaviour, and intentions are influenced by subjective norms, which correspond to perceived social influences. Our results suggest that using social eating norms could be an effective way to enhance the likelihood that individuals implement recommendations. For example, communication about the healthy eating behaviours of a target population could be an effective way to set up new social eating norms and sustainably improve individuals’ behaviours [44,46]. More generally, making changes alongside family members or relatives out of home might be encouraged, as they may be more likely to be maintained.

### 4.4. Limitations

This study is based on self-reported consumption and perceptions of social eating norms, which are subject to error and bias. Another methodological limitation is that lockdown and post-lockdown consumptions and perceived social eating norms were recorded at the same period, in November 2020. Hence, it may have been difficult for participants to recall the specific changes that took place during each period. This might have led to an overestimation of the maintenance of changes. Despite this inevitable methodological limitation due to the suddenness of the COVID-19 pandemic, this specific situation gave us a unique window to study the relationship between social eating norms and dietary habits, in real-life conditions with a large sample.

## 5. Conclusions

To our knowledge, this study is the first to examine associations between the perception of eating social norms and the initiation and maintenance of changes to dietary habits that took place during the first lockdown in France. We found a significant relationship between the perceptions of changes made during lockdown by household members and relatives out of home, but not by the general population, as well as the dietary changes initiated by the participants, for all food categories assessed. The likelihood of maintaining changes was also significantly increased by the positive perception of changes made during lockdown by household members. This study also provides novel results on the maintenance of these changes, as we found that around 65% of the individuals who reported changes also said they had maintained those changes 5 months after the end of the lockdown. To conclude, this study highlights a potential role for perceived social eating norms, especially those regarding household members and non-household relatives, in both the implementation and the maintenance of dietary changes that took place during the first COVID-19 lockdown in France. More generally, these results confirm that social eating norms have a powerful influence on individuals’ behaviours and suggest that the probability of dietary changes being maintained is enhanced when they are established in line with the normative behaviour of familiar others.

## Figures and Tables

**Table 1:** Categorisation of usual food items into two categories based on the French National Health Agency recommendations (Ministère des Solidarités et de la Santé, 2019).

Foods to Increase	Foods to Decrease
Fruits and vegetables Pulses Nuts and seeds Homemade meals	Meat Cold meats Sodas Alcohol Biscuits and sweets

**Table 2:** Construction of the variable “maintenance of changes” based on reported consumption during and after lockdown compared to before lockdown.

		Reported Consumption during Lockdown Compared to before Lockdown		
		Increase	Decrease	Same
Reported consumption after lockdown compared to before lockdown	Increase	Maintenance of increase	No maintenance	Excluded
	Decrease	No maintenance	Maintenance of decrease	Excluded
	Same	No maintenance	No maintenance	Excluded

**Table 3:** Comparison of demographic information, education level, and employment status between included and excluded individuals.

	Total Population	Included	Excluded	Comparison Included vs. Excluded (*p*-Value)
**Sex**	909 W (54%) 785 M	567 W (56%) 411 M	342 W (50%) 344 M	*p* = 0.01
**Age** (years)	47.6 (±14.8)	45.6 (±14.9)	50.6 (±14.1)	*p* < 0.001
**BMI** (kg·m^2^)	25.1 (±4.9)	24.9 (±4.7)	25.4 (±5.3)	*p* = 0.07
**Education level**				
No diploma		1%	2%	NS
Secondary Education	2%	3%	4%	NS
Professional diploma	3%	12%	23%	*p* < 0.001
High school	17%	22%	22%	NS
High school + 2 year diploma	23%	24%	21%	NS
High school + 3 year diploma	23%	16%	14%	NS
High school + 5 year diploma (equivalent to a master’s degree)	15%	19%	11%	*p* < 0.001
High school + 8 year diploma (equivalent to a doctoral degree)	1%	2%	1%	NS
**Employment status**				
Labourer	5%	5%	8%	NS
Employee	27%	28%	26%	NS
Executive	14%	17%	10%	*p* < 0.001
Farmer	<1%	<1%	<1%	NS
Artisan, merchant, entrepreneur	3%	3%	2%	NS
Intermediate profession	17%	19%	15%	NS
Retired	21%	17%	28%	*p* < 0.001
Unemployed	11%	11%	14%	NS

*t*-Tests were used to compare the age and BMI; chi-square tests and post hoc chi-square tests were used to compare the distribution of sex, education level, and profession. NS = nonsignificant; W = women; M = men.

**Table 4:** Results of multinomial logistic regressions evaluating the influence of perceived lockdown social eating norms on changes of consumption for the two food categories.

Social Eating Norm Type	Social Eating Norm Perception	Food Category	Changes in Consumption	OR and CI	*p*-Value
**Household**	Yes +	To increase	Increase	2.07 (1.48–2.89)	<0.001
		To decrease	Decrease	2.13 (1.50–3.02)	<0.001
			Increase	1.70 (1.08–2.68)	0.02
	Yes −	To increase	None	--	NS
		To decrease	Increase	5.55 (1.61–19.09)	0.006
**Non-Household Relatives**	Yes +	To increase	Increase	1.61 (1.11–2.33)	0.01
		To decrease	None	--	NS
	Yes −	To Increase	None	--	NS
		To decrease	None	--	NS
**General Population**	*No significant results*				

OR = odds ratios; CI = confidence interval at 95%; *p*-value = 5%; NS = nonsignificant; Yes + = perception of positive changes; Yes − = perception of negative changes.

**Table 5:** Results of multinomial logistic regressions evaluating the influence of lockdown social eating norms on the maintenance on changes for the two food categories.

Social Eating Norm Type	Social Eating Norm Perception	Food Category	Maintained Pattern	OR and CI	*p*-Value
**Household**	Yes +	To increase	Increase	1.65 (1.05–2.58)	0.03
		To decrease	None	--	NS
	Yes −	To increase	None	--	NS
		To decrease	None	--	NS
**Relatives**	*No significant results*				
**General Population**	*No significant results*				

OR = odds ratios; CI = confidence interval at 95%; *p*-value= 5%; NS= non-ignificant; Yes + = perception of positive changes; Yes − = perception of negative changes.

## Data Availability

Data available in a publicly accessible repository. The data presented in this study are openly available in OSF at https://mfr.osf.io/render?url=https://osf.io/upxvb/?direct%26mode=render%26action=download%26mode=render.

## Supplementary Materials

The following are available online at https://www.mdpi.com/article/10.3390/foods10112745/s1, Annex S1: Consent questionnaire in French, Annex S2: Questionnaire in French, Annex S3: Changes in food choice motives during and after lockdown compared to before. Table S1: Complete statistical results of all the models for Hypotheses 1 and 2.
